# Yeast GH30 Xylanase from *Sugiyamaella lignohabitans* Is a Glucuronoxylanase with Auxiliary Xylobiohydrolase Activity §

**DOI:** 10.3390/molecules27030751

**Published:** 2022-01-25

**Authors:** Katarína Šuchová, Andrej Chyba, Zuzana Hegyi, Martin Rebroš, Vladimír Puchart

**Affiliations:** 1 Institute of Chemistry, Slovak Academy of Sciences, Dúbravská Cesta 9, 845 38 Bratislava, Slovakia; Andrej.Chyba@savba.sk (A.C.); Vladimir.Puchart@savba.sk (V.P.); 2 Institute of Biotechnology, Faculty of Chemical and Food Technology, Slovak University of Technology in Bratislava, Radlinského 9, 812 37 Bratislava, Slovakia; zuzana.hegyi@stuba.sk (Z.H.); martin.rebros@stuba.sk (M.R.)

**Keywords:** xylanase, glucuronoxylanase, xylobiohydrolase, xylan, glycoside hydrolase family 30, GH30-7 subfamily, *Sugiyamaella lignohabitans*, yeast

## Abstract

Xylanases are the enzymes that catalyze the breakdown of the main hemicellulose present in plant cell walls. They have attracted attention due to their biotechnological potential for the preparation of industrially interesting products from lignocellulose. While many xylanases have been characterized from bacteria and filamentous fungi, information on yeast xylanases is scarce and no yeast xylanase belonging to glycoside hydrolase (GH) family 30 has been described so far. Here, we cloned, expressed and characterized GH30 xylanase *Sl*Xyn30A from the yeast *Sugiyamaella lignohabitans*. The enzyme is active on glucuronoxylan (8.4 U/mg) and rhodymenan (linear β-1,4-1,3-xylan) (3.1 U/mg) while its activity on arabinoxylan is very low (0.03 U/mg). From glucuronoxylan *Sl*Xyn30A releases a series of acidic xylooligosaccharides of general formula MeGlcA^2^Xyl_n_. These products, which are typical for GH30-specific glucuronoxylanases, are subsequently shortened at the non-reducing end, from which xylobiose moieties are liberated. Xylobiohydrolase activity was also observed during the hydrolysis of various xylooligosaccharides. *Sl*Xyn30A thus expands the group of glucuronoxylanases/xylobiohydrolases which has been hitherto represented only by several fungal GH30-7 members.

## 1. Introduction

Endo-β-1,4-xylanases (EXs, EC 3.2.1.8) are main xylan depolymerizing enzymes cleaving the polysaccharide backbone to xylooligosaccharides (XOs) of various lengths. Most EXs are classified in glycoside hydrolase (GH) families 10 and 11 (www.cazy.org, accessed on 1 December 2021) [1]; however, they are also found in GH families 5, 8, 30, 43, 98 and 141. During the last few years, great attention has been paid to the EXs from GH30 family [2,3,4,5,6,7,8,9,10,11,12,13]. Prokaryotic EXs are grouped into subfamily GH30-8, while eukaryotic xylanases are members of GH30-7 subfamily. Catalytic properties of the GH30-8 subfamily enzymes are quite uniform and most of them are specific glucuronoxylanases (EC 3.2.1.136) requiring glucuronic or 4-*O*-methyl-glucuronic acid (MeGlcA) substitution of the main chain for their action [13,14,15]. On the other hand, catalytic properties of the GH30-7 subfamily representatives are diverse and include specific glucuronoxylanases, xylobiohydrolases, non-specific endoxylanases, endoxylanases/xylobiohydrolases and xylanases releasing xylose from the reducing end of the substrate [11,12]. All characterized GH30-7 xylanases come from filamentous fungi and so far no yeast GH30 EX has been described.

Compared to bacterial and fungal xylanases, yeast xylanases have not been studied so extensively, presumably due to much lower levels of xylanase production by the yeasts. The best characterized yeast xylanases are from yeast-like fungus *Aureobasidium pullulans* [16,17] and from several *Cryptococcus* species [18,19,20], but EXs were purified and characterized also from *Pichia* (*Scheffersomyces*), *Pseudozyma* *and Blastobotrys* genera [21,22,23]. All of them belong to either the GH10 or GH11 family. Bioinformatic mining in 332 yeast genomes from the phylum *Ascomycota* has revealed only a few putative yeast xylanases: one from the GH11 family (*Blastobotrys mokoenii*), five from the GH10 family (two from *Spencermartinsiella europaea,* two from *Sugiyamaella lignohabitans* and one from *B. peoriensis*) and three from the GH30-7 subfamily (*Sp. europaea, Su lignohabitans, B. mokoenii*) [23].

The genus *Sugiyamaella*, which belongs to the family *Trichomonascaceae*, was established by Kurtzman and Robnett [24]. The members of *Sugiyamaella* clade are mostly found in a wood environment. *Sugiyamaella* (*Candida*) *lignohabitans* strains were isolated from tenebrionid beetles inhabiting a rotten log or from decayed wood [25]. A total of 16 yeast isolates belonging to the genus *Sugiyamaella* recovered from rotting wood and sugarcane bagasse samples in different Brazilian regions were studied in relation to d-xylose fermentation, xylitol production, and xylanase activities [26]. All of them exhibited xylanase activity and almost all were able to produce ethanol and xylitol. Among them, *S. lignohabitans* exhibited the highest xylanase activity and the best xylitol yield and productivity after 24 h [26]. This yeast has been also described as a host for the production of organic acids from lignocellulosic hydrolysates [27]. Yeast strains able to degrade polysaccharides and simultaneously convert their constituents to desired products are interesting for possible applications in lignocellulosic biorefining.

To expand the knowledge on yeast xylanases, we have cloned, expressed and characterized GH30 xylanase *Sl*Xyn30A from *S. lignohabitans*. It was found to be a glucuronoxylanase with auxiliary xylobiohydrolase activity.

## 2. Results

### 2.1. Sequence Analysis of SlXyn30A

*Sl*Xyn30A (GenBank: ANB12318) is composed of 470 amino acids, including signal peptide which is 16 amino acids long. The mature protein has a theoretical molecular mass of 49,539 Da. BLAST search (https://blast.ncbi.nlm.nih.gov/Blast.cgi, accessed on 2 November 2021) showed the highest similarity (68.4%, identity 53.7%) to a putative GH30 xylanase from *Lineolata rhizophorae* (GenBank: KAF2460202.1). Of the characterized GH30 enzymes, *Sl*Xyn30A showed the highest similarity (65.9%) and identity (47.7%) to xylanase C from *Talaromyces purpureogenus*, but other hits showed a similar level of homology and identity: glucuronoxylanase/xylobiohydrolase *Tt*Xyn30A from *Thermothelomyces thermophila* (65.5% similarity, 47.5% identity), glucuronoxylanase/xylobiohydrolase *Tc*Xyn30B from *Talaromyces cellulolyticus* (63.4% similarity, 42.1% identity), xylanase XylD from *Bispora* (61.9% similarity, 42.7% identity), and glucuronoxylanase *Tr*XynVI from *Trichoderma reesei* (59.7% similarity, 44.3% identity).

**Figure 1:**
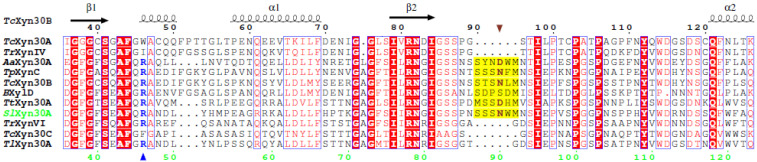
Amino acid sequence alignment of *Sl*Xyn30A with GH30-7 xylanases *Talaromyces cellulolyticus Tc*Xyn30B (GAM36763), *Tc*Xyn30C (GAM40414.1), *Tc*Xyn30A (GAM43270), *Thermothelomyces thermophila Tt*Xyn30A (AEO55025), *Talaromyces purpureogenus* (*Penicillium purpurogenum*) *Tp*XynC (AKH40280), *Bispora* sp. *B*XylD (ADG62369.1), *Trichoderma reesei Tr*XynVI (EGR45006.1), *T. reesei Tr*XynIV (AAP64786.1), *Acremonium alcalophilum Aa*Xyn30A [7], and *Talaromyces leycettanus Tl*Xyn30A [10]. The secondary structure elements and numbering of *Tc*Xyn30B are shown on top, numbering of *Sl*Xyn30A is at the bottom. Arginine suggested to be responsible for the recognition of MeGlcA substitution is shown in blue and is marked by a blue up triangle. Longer β2-α2 loop in xylobiohydrolases is highlighted in yellow and the residue interacting with Xyl*p* moiety occupying the −2a subsite is brown and marked by a brown down triangle.

Based on amino acid sequence alignment (Figure S1, Supplementary Materials), two catalytic residues were identified: Glu199 as an acid/base and Glu292 as a nucleophile. The alignment also revealed that *Sl*Xyn30A displays structural features of the GH30-7 enzymes including a longer β2-α2 loop, a lack of α6-helix, and a presence of β-strands β8A and β8B [12]. Moreover, *Sl*Xyn30A contains several structural elements typical for GH30-7 glucuronoxylanases/xylobiohydrolases (Figure 1). The first is a presence of Arg46 which was suggested to be responsible for a MeGlcA recognition and glucuronoxylanase activity of *Tc*Xyn30B [4]. Although the 3D structure of another GH30-7 glucuronoxylanase, *Tt*Xyn30A, did not confirm such a role of the arginine, mutational studies of *Tt*Xyn30A indicated its importance [9]. The second structural feature considered to be responsible for xylobiohydrolase activity is the length and amino acid sequence of the β2-α2 loop. This loop is of the same length in *Sl*Xyn30A, *Tc*Xyn30B and *Tt*Xyn30A, and is longer than in other GH30-7 enzymes (e.g., *Tr*XynVI; Figure 1). Asn93 in *Tc*Xyn3B and Asp78 in *Tt*Xyn30A in this loop were shown to interact with Xyl*p* residue accommodated in the −2a subsite [4,9]. The corresponding residue in *Sl*Xyn30A is Asn90 which may play a similar role.

### 2.2. Recombinant Strain Selection

Four clones with integrated pPICZαA vector carrying the *Slxyn30A* gene were selected from the ZeocinTM (250 mg/L) plate. The clones were individually cultivated in shake flasks, and after 120 h of induction, supernatant was screened by SDS-PAGE electrophoresis for the presence of 50 kDa protein which should correspond to a mature enzyme. The expected protein was confirmed in three transformants (Figure S2). The molecular mass of the enzyme was, however, a little bit higher (58 kDa), presumably due to glycosylation. According to NetNGlyc server, the amino acid sequence of *Sl*Xyn30A contains 4 potential N-glycosylation sites (N86, N114, N252, N306). The first one is conserved in *Tc*Xyn30B where is actually glycosylated [2]. Three transformants were then tested for xylanase activity and the transformant with the highest activity (transformant 4) was used for the determination of the catalytic properties of *Sl*Xyn30A.

### 2.3. Thermal and pH Optima and Stability

**Figure 2:**
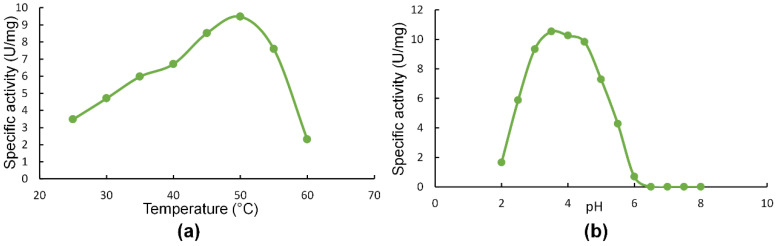
Effect of temperature (**a**) and pH (**b**) on activity of *Sl*Xyn30A.

*Sl*Xyn30A showed a temperature optimum of 50 °C, showing 71% and 24% of maximal activity at 40 °C and 60 °C, respectively (Figure 2a). pH optimum was around 3.5 and the enzyme was active in acidic range of pH, keeping only 6.6% of the maximal activity at pH 6 (Figure 2b). The enzyme was stable at temperatures up to 50 °C, while at 60 °C it completely lost its activity within 30 min.

### 2.4. Hydrolysis of Polysaccharides

**Figure 3:**
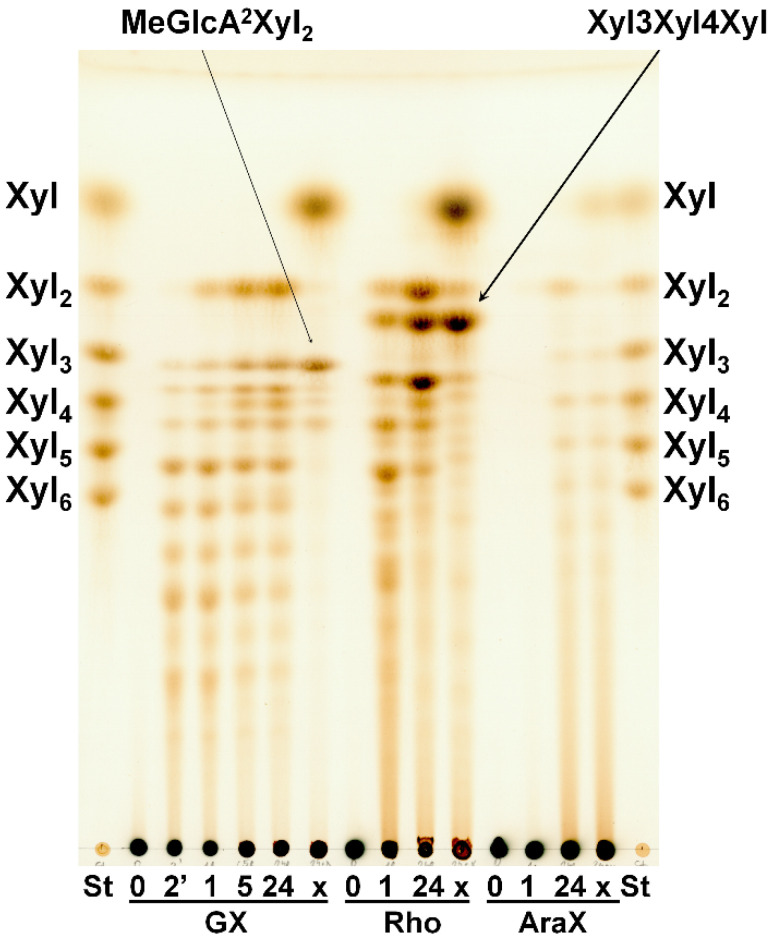
TLC analysis of hydrolysis products released from GX, Rho and AraX by *Sl*Xyn30A after 2 min, 1 h, 5 h, 24 h and after treatment with β-xylosidase (x). St—standards of linear XOs.

*Sl*Xyn30A showed the highest specific activity on glucuronoxylan (GX) (8.4 U/mg), while activity on linear β-1,3-1,4-xylan (rhodymenan, Rho) was about 2.5 times lower (3.1 U/mg). Specific activity on arabinoxylan was extremely low (0.03 U/mg). TLC analysis of hydrolysis products showed that GX was initially cleaved to a series of acidic xylooligosaccharides (XOs) (Figure 3) which were partly shortened after a prolonged incubation.

**Figure 4:**
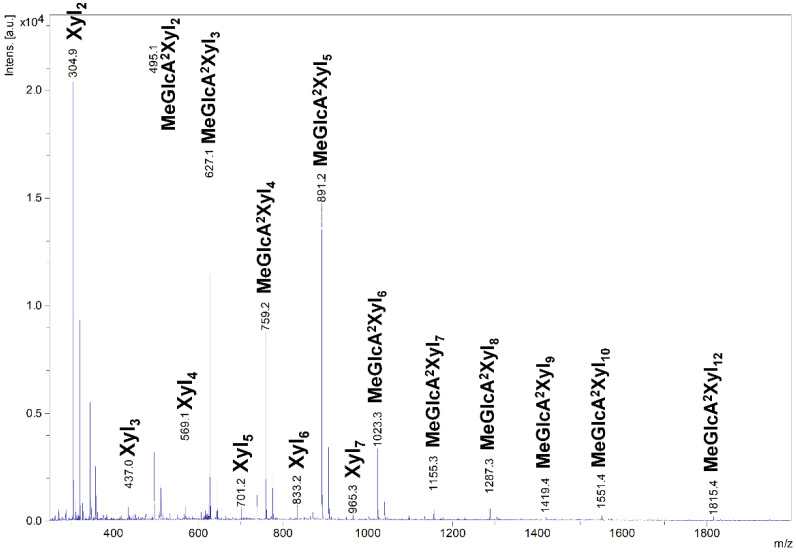
MALDI-ToF MS analysis of 5-day hydrolysate of GX by *Sl*Xyn30A.

The acidic XOs shortening was accompanied by xylobiose (Xyl_2_) formation, which was detectable after 1 h, indicating xylobiohydrolase activity of the enzyme. Later, after 5 h, xylotetraose (Xyl_4_) also appeared as a result of transglycosylation reaction. After an application of β-xylosidase to 24 h hydrolysate, the acidic XOs were hydrolyzed to MeGlcA^2^Xyl_2_ which indicates that all acidic XOs had MeGlcA substitution on the second xylopyranosyl (Xyl*p*) residue from the reducing end. This mode of GX hydrolysis is typical for the GH30 glucuronoxylanases [13,14,15]. Glucuronoxylanase activity accompanied by xylobiohydrolase activity has been already described for other GH30-7 enzymes *Tc*Xyn30B and *Tt*Xyn30A [2,3]. Our results indicate that *Sl*Xyn30A might also be a glucuronoxylanase/xylobiohydrolase. However, the acidic XOs in the 24 h hydrolysate were not shortened exclusively to MeGlcA^2^Xyl_2_ and MeGlcA^2^Xyl_3_, as was observed in the case of *Tt*Xyn30A [3], but longer acidic products remained in the hydrolysate even after 5-day incubation or after an addition of fresh enzyme. In this regard, *Sl*Xyn30A resembles much more *Tc*Xyn30B than *Tt*Xyn30A. MALDI-ToF analysis of the 5-day hydrolysate confirmed the presence of a broad spectrum of acidic XOs from MeGlcAXyl_2_ to MeGlcAXyl_12_ (MeGlcAXyl_2_—MeGlcAXyl_5_ prevailing) (Figure 4). Xyl_2_ was predominant neutral XO but traces of longer XOs up to DP10 were also observed. Kinetic parameters determined for GX were K_m_ 16.8 mg/mL, k_cat_ 20.2 s^−1^ and k_cat_/K_m_ 1.2 mL/mg·s. Rho was hydrolyzed to a mixture of β-1,4-linked XOs and β-1,3-1,4-XOs, among which the most predominant were Xyl_2_, Xyl_4_, Xylβ1-3Xylβ1-4Xyl and Xylβ1-4Xylβ1-3Xylβ1-4Xyl (Figure 3). β-Xylosidase added to the 24 h-hydrolysate of Rho cleaved all β-1,4-linked XOs to Xyl and all the β-1,3-1,4-linked XOs to Xylβ1-3Xylβ1-4Xyl. The final extent of hydrolysis seems to be higher for Rho than GX. Hydrolysis of AraX was very weak (Figure 3). In this case, Xyl_2_ was a predominant product accompanied by a few Ara-substituted XOs, most probably having the Ara substitution on the non-reducing end (they were not attacked by β-xylosidase).

### 2.5. Hydrolysis of Oligosaccharides

**Figure 5:**
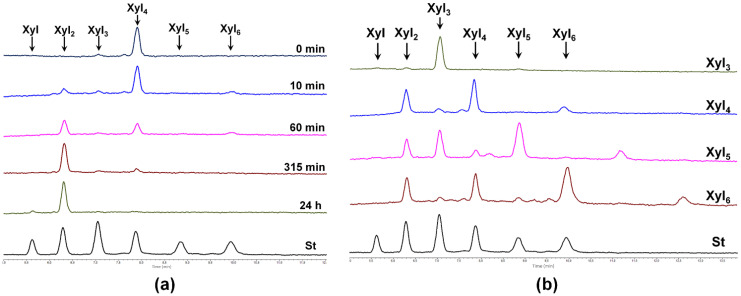
HPLC analysis of hydrolysis of XOs by *Sl*Xyn30A. (**a**) Time course of Xyl_4_ hydrolysis; (**b**) Analysis of hydrolysis products generated from Xyl_3_-Xyl_6_ after 30 min of reaction.

**Figure 6:**
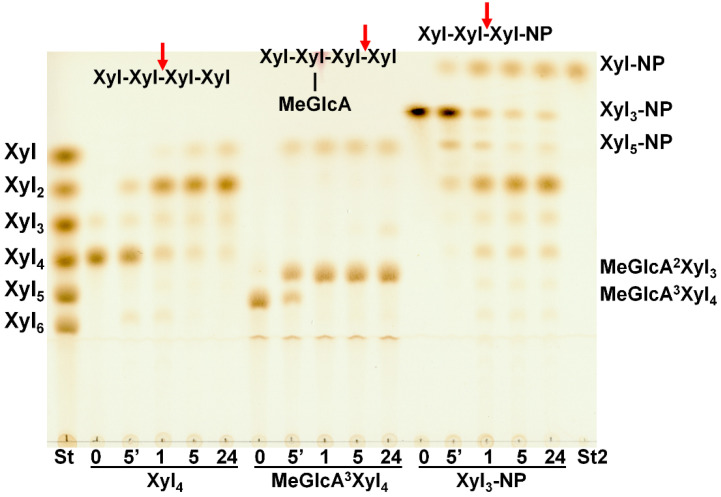
TLC analysis of hydrolysis products released from Xyl_4_, MeGlcA^3^Xyl_4_ and Xyl_3_-NP by *Sl*Xyn30A after 5 min, 1 h, 5 h and 24 h. St—standards of linear XOs, St2—standard of 4-nitrophenyl β-d-xylopyranoside.

Xylobiohydrolase activity of *Sl*Xyn30A was also observed on various XOs. The main hydrolysis product released from Xyl_4_ was Xyl_2,_ but Xyl_6_ was also formed, presumably through transglycosylation reaction (Figure 5a). Xyl_5_ was mainly cleaved to Xyl_2_ and Xyl_3_, and Xyl_6_ to Xyl_2_ and Xyl_4_. In both cases, the transglycosylation products with DP higher by two than the substrate were observed during the early stages of reaction (Figure 5b). Xyl_3_ was the worst substrate, being cleaved to Xyl_2_ and Xyl only after a prolonged incubation. Transglycosylation products were also formed during the action of *Sl*Xyn30A on 4-nitrophenyl glycosides of β-1,4-xylobiose (Xyl_2_-NP) and β-1,4-xylotriose (Xyl_3_-NP). Xyl_2_ release was accompanied by a liberation of 4-nitrophenol from Xyl_2_-NP and 4-nitrophenyl xyloside from Xyl_3_-NP. This mode of action has unambiguously confirmed that xylobiose moiety is released from the non-reducing end of the substrates. Compared to linear β-1,4-XOs and the corresponding NP-glycosides, MeGlcA-substituted XOs of the same DP were cleaved faster (Figure 6). About 60% of MeGlcA^3^Xyl_4_ was hydrolyzed after 5 min of the reaction, when only about 10% of Xyl_4_ or Xyl_3_-NP were converted. Specific activities were 0.037 U/mg for Xyl_3_, 0.222 U/mg for Xyl_4_, 1.9 U/mg for MeGlcA^3^Xyl_4_ and 56.7 U/mg for MeGlcA^3^Xyl_3_ showing the preference of the enzyme for MeGlcA-substituted substrates. It should be noted that the transglycosylation reaction was not observed during the processing of the acidic XOs MeGlcA^3^Xyl_4_ and MeGlcA^3^Xyl_3_.

**Figure 7:**
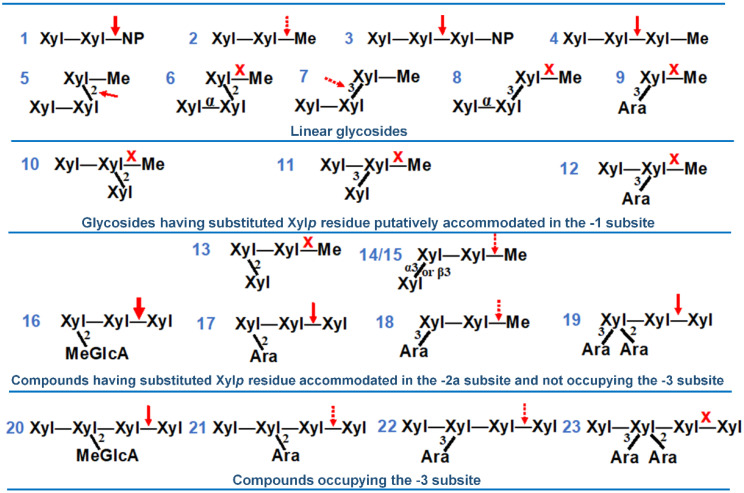
Various XOs tested as the substrates for *Sl*Xyn30A. The site of cleavage is denoted by an arrow, X marks compounds which were not attacked.

The activity of *Sl*Xyn30A was further tested on oligosaccharides containing β-1,3- or β-1,2-linkages or arabinosyl substitution (Figure 7, compounds **1**–**23**). The enzyme was able to slowly release methanol from methyl β-1,4-xylobioside (**2**) indicating that Xyl*p* unit accommodated in the +1 subsite is not indispensable for enzyme activity. Methyl β-1,4-xylotrioside (**4**) was hydrolyzed much faster than (**2**), and exclusively to Xyl_2_ and Xyl-Me in accordance with xylobiohydrolase activity of the enzyme. *Sl*Xyn30A was able to cleave very slowly also β-1,2- and β-1,3-linkages in X4X2XMe (**5**) and X4X3XMe (**7**), the former being cleaved faster but significantly slower than (**4**). However, if Xyl*p* residues at the non-reducing end are connected by α-1,4-linkage, the substances (**6**,**8**) are not hydrolyzed. The compounds, in which the substitution would occur on Xyl*p* unit accommodated in the −1 subsite, were not hydrolyzed (**9**–**12**). On the other hand, some substitutions of Xyl*p* in the −2a subsite were tolerated. The tolerance was influenced by three factors: (1) position of the decoration (2 and/or 3); (2) the nature of the substituent; and (3) whether the compound was further elongated at the non-reducing end, i.e., it carried additional Xyl*p* residue that was accommodated in the subsite −3. If the Xyl*p* unit accommodated in the −2a subsite is decorated at position 2 by the MeGlcA, the substrate (MeGlcA^3^Xyl_3_, **16**) is readily hydrolyzed. When the MeGlcA substitution is replaced by α-l-arabinofuranose, the substrate (A2X4X4X, **17**) is slowly attacked, but a change for β-d-xylopyranose (X2X4XMe, **13**) caused a resistance to the enzyme attack. The tolerance at position 3 is greater since A3X4XMe (**18**) and X3X4XMe (**14**) as well as Xα3X4XMe (**15**) were slowly processed. *Sl*Xyn30A was also able to cleave the substrate (**19**) with doubly 2,3-*O*-arabinosylated Xyl*p* residue accommodated in the −2a subsite. However, if the compounds with the substitution on Xyl*p* residue in the −2a subsite are by one Xyl*p* longer at the non-reducing end (i.e., they occupy also the −3 subsite), the hydrolysis is slowed down (**20**–**22**) or even abolished (**23**).

## 3. Discussion

Many xylanases have been isolated and characterized from bacteria and filamentous fungi while a number of characterized yeast xylanases is limited and no yeast GH30 xylanase has been described so far. Catalytic properties of eukaryotic GH30 xylanases belonging to GH30-7 subfamily, where *Sl*Xyn30A is also grouped, are diverse. It was, therefore, interesting to determine the specificity of the yeast xylanase, which may be related to a biotechnological potential of the yeast due to its reported ability to convert xylose to xylitol, ethanol or organic acids [26,27]. The GH30 xylanase *Sl*Xyn30A was cloned, expressed, and characterized. *Sl*Xyn30A showed the highest amino acid similarity to glucuronoxylanases/xylobiohydrolases *Tt*Xyn30A and *Tc*Xyn30B. *Sl*Xyn30A, similarly to these fungal enzymes, contains Arg46 which was shown to play a role in MeGlcA recognition [4,9]. Another aspect of similarity between the three enzymes is the exact length of β2-α2 loop, which may affect the occupation of the −3 subsite. Moreover, Asn90 in this loop corresponds to Asn93 in *Tc*Xyn3B and Asp78 in *Tt*Xyn30A which are supposed to play a role in xylobiohydrolase activity of the GH30-7 glucuronoxylanases [4,9]. All these structural features of *Sl*Xyn30A are in accordance with its biochemical properties. Glucuronoxylan was the best substrate among the heteroxylans studied and during its hydrolysis, the MeGlcA residue was accommodated in the −2b subsite of the enzyme, yielding acidic XOs of general formula MeGlcA^2^Xyl_n_. Such a hydrolysis of GX is typical for GH30 glucuronoxylanases [13,14,15]. Later, the acidic XOs were partially shortened by *Sl*Xyn30A through a liberation of Xyl_2_ from the non-reducing end, similarly to GH30 xylobiohydrolases [7,8] and glucuronoxylanses/xylobiohydrolases [2,3]. The shortening of the acidic XOs was not complete and the aldouronic acids of a medium size persisted in the *Sl*Xyn30A hydrolysate. From this point of view, *Sl*Xyn30A most resembles *Tc*Xyn30B, which shows an essentially identical end-stage hydrolysis profile [2], and slightly differs from *Tt*Xyn30A, which liberated the acidic XOs and Xyl_2_ simultaneously, and Xyl_2_, MeGlcA^2^Xyl_2_ and MeGlcA^2^Xyl_3_ were the only final products of GX hydrolysis [3]. In addition, kinetic parameters of *Sl*Xyn30A on GX are also similar to those of *Tc*Xyn30B [2].

The xylobiohydrolase activity of *Sl*Xyn30A was even more pronounced during the hydrolysis of rhodymenan. It was reflected in an accumulation of not only Xyl_2_, but also isomeric xylotriose Xylβ1-3Xylβ1-4Xyl and isomeric xylotetraose Xylβ1-4Xylβ1-3Xylβ1-4Xyl. The release of Xylβ1-3Xylβ1-4Xyl is in agreement with the ability of *Sl*Xyn30A to cleave X3X4XMe (Figure 7, **14**) to X3X4X and methanol. Hydrolysis of X4X3XMe is very slow (not finished after 145 h of hydrolysis) indicating a very limited ability of the enzyme to hydrolyze β-1,3-xylosidic linkage. This is in accordance with the presence of Xylβ1-4Xylβ1-3Xylβ1-4Xyl in 24 h hydrolysate of Rho. In contrast, xylobiohydrolases *Aa*Xyn30A and *Hc*Xyn30A are able to cleave β-1,3-linkages much more efficiently and therefore Xylβ1-4Xylβ1-3Xylβ1-4Xyl was not accumulated but cleaved to two molecules of Xyl_2_ in their hydrolysates [7,8].

Linear β-1,4-XOs were processed by *Sl*Xyn30A in the same way as was described for *Tc*Xyn30B and *Tt*Xyn30A [2,3]. Xyl_2_ was the main product and XOs longer by two xylose units were formed via transglycosylation. For *Sl*Xyn30A, Xyl_3_ was a much worse substrate than Xyl_4_, suggesting that an occupation of the +2 subsite has a significant positive effect on the enzyme activity.

The ability of *Sl*Xyn30A to recognize MeGlcA substitution was confirmed by a comparison of specific activities on linear XOs and the corresponding XOs decorated by MeGlcA. Specific activity on MeGlcA^3^Xyl_3_ was about 1500 times higher than on Xyl_3_. On the other hand, MeGlcA^3^Xyl_4_ was only about 8 times better substrate than Xyl_4_. MeGlcA^3^Xyl_3_ was hydrolyzed about 30 times faster than MeGlcA^3^Xyl_4_. These data clearly indicate the preference of the enzyme for the MeGlcA-decorated substrates and for the substrates not occupying the −3 subsite of the enzyme. The latter preference was confirmed also during the hydrolysis of various methyl glycosides and arabinoxylooligosaccharides when elongation of the XO chain to the −3 subsite of the enzyme (Figure 7, **16** vs. **20**, **17** vs. **21**, **18** vs. **22**, **19** vs. **23**) caused a slowdown or an abolishment of the reaction. The evaluation of these compounds allowed us to draw the following conclusions on the requirement of *Sl*Xyn30A on the structure of the substrates. First, *Sl*Xyn30A does not tolerate any substitution on Xyl*p* residue accommodated in the −1 subsite. Xylose residue accommodated in the −2a subsite may be substituted at position 2 by MeGlcA, which improves the activity, and by α-l-arabinofuranose but not by β-d-xylopyranose. Position 3 may be both arabinosylated and xylosylated, but the activity on such substrates is lower compared to unsubstituted XOs. The elongation of the substrate main chain that results in an occupation of the −3 subsite may dramatically decrease the hydrolysis rate.

## 4. Materials and Methods

### 4.1. Substrates, Standards and Enzymes

Beechwood 4-*O*-methylglucuronoxylan (GX), aldotetraouronic acid MeGlcA^3^Xyl_3_ and aldopentaouronic acid MeGlcA^3^Xyl_4_ were prepared as described earlier [28,29]. Rhodymenan, an algal linear β-1,3-β-1,4-xylan from *Palmaria palmata*, was a kind gift of Prof. M. Claeyssens (University of Ghent, Ghent, Belgium). Wheat arabinoxylan (Ara:Xyl 38:62, medium viscosity), 4-nitrophenyl glycosides of xylose, xylobiose and xylotriose, linear β-1,4-xylooligosaccharides (Xyl_2_-Xyl_6_) and arabinoxylooligosaccharides (Figure 7) A2X4X4X α-l-Ara*f*-1,2-β-d-Xyl*p*-1,4-β-d-Xyl*p*-1,4-d-Xyl, (**17**), A3[A2]X4X4X (α-l-Ara*f*-1,3-[α-l-Ara*f*-1,2]-β-d-Xyl*p*-1,4-β-d-Xyl*p*-1,4-d-Xyl, (**19**), X4[A3]X4X4X (β-d-Xyl*p*-1,4-[α-l-Ara*f*-1,3]-β-d-Xyl*p*-1,4-β-d-Xyl*p*-1,4-d-Xyl, (**18**), X4[A2][A3]X4X4X (β-d-Xyl*p*-1,4-[α-l-Ara*f*-1,2][α-l-Ara*f*-1,3]-β-d-Xyl*p*-1,4-β-d-Xyl*p*-1,4-d-Xyl, (**23**) and a mixture of X4[A3]X4X4X and X4[A2]X4X4X (β-d-Xyl*p*-1,4-[α-l-Ara*f*-1,2]-β-d-Xyl*p*-1,4-β-d-Xyl*p*-1,4-d-Xyl, (**22**,**21**) were purchased from Megazyme International (Bray, Ireland). Methyl glycosides of (arabino)xylooligosaccharides (Figure 7)—A3XMe (α-l-Ara*f*-1,3-β-d-Xyl*p*-*O*-Me, (**9**), X4[A3]XMe (β-d-Xyl*p*-1,4-[α-l-Ara*f*-1,3]-β-d-Xyl*p*-*O*-Me, (**12**), X4XMe (β-d-Xyl*p*-1,4-β-d-Xyl*p*-*O*-Me, (**2**), X4X4XMe (β-d-Xyl*p*-1,4-β-d-Xyl*p*-1,4-β-d-Xyl*p*-*O*-Me, (**4**), X4X3XMe (β-d-Xyl*p*-1,4-β-d-Xyl*p*-1,3-β-d-Xyl*p*-*O*-Me, (**7**), X4X2XMe (β-d-Xyl*p*-1,4-β-d-Xyl*p*-1,2-β-d-Xyl*p*-*O*-Me, (**5**), X3X4XMe (β-d-Xyl*p*-1,3-β-d-Xyl*p*-1,4-β-d-Xyl*p*-*O*-Me, (**14**), Xα3X4XMe (α-d-Xyl*p*-1,3-β-d-Xyl*p*-1,4-β-d-Xyl*p*-*O*-Me, (**15**), X2X4XMe (β-d-Xyl*p*-1,2-β-d-Xyl*p*-1,4-β-d-Xyl*p*-*O*-Me, (**13**), X4[X3]XMe (β-d-Xyl*p*-1,4-[β-dxyl*p*-1,3]-β-d-Xyl*p*-*O*-Me, (**11**), X4[X2]XMe (β-d-Xyl*p*-1,4-[β-d-Xyl*p*-1,2]-β-d-Xyl*p*-*O*-Me, (**10**), Xα4X3XMe (α-d-Xyl*p*-1,4-β-d-Xyl*p*-1,3-β-d-Xyl*p*-*O*-Me, (**8**), Xα4X2XMe (α-d-Xyl*p*-1,4-β-d-Xyl*p*-1,2-β-d-Xyl*p*-*O*-Me, (**6**)—were synthesized previously [30,31,32,33,34,35] and were generously supplied by Dr. Ján Hirsch (Institute of Chemistry, Slovak Academy of Sciences, Bratislava, Slovakia). β-Xylosidase was a recombinant *Aspergillus niger* enzyme from GH3 family expressed in *Saccharomyces cerevisiae* [36].

### 4.2. Amino Acid Sequence Comparison

Amino acid sequence of *Sl*Xyn30A (GenBank: ANB12318) was aligned with amino acid sequences of *Talaromyces cellulolyticus Tc*Xyn30B (GAM36763), *Tc*Xyn30C (GAM40414.1), *Tc*Xyn30A (GAM43270), *Thermothelomyces thermophila Tt*Xyn30A (AEO55025), *Talaromyces purpureogenus* (*Penicillium purpurogenum*) *Tp*XynC (AKH40280), *Bispora* sp. *B*XylD (ADG62369.1), *Trichoderma reesei Tr*XynVI (EGR45006.1), *T. reesei Tr*XynIV (AAP64786.1), *Acremonium alcalophilum Aa*Xyn30A [7], and *Talaromyces leycettanus Tl*Xyn30A [10] using Clustal Omega server [37] and visualized by ESPript server [38].

### 4.3. Recombinant Strain Preparation

In this work, *P. pastoris* KM71H (MutS strain) was used. The strain preparation and cultivation conditions for production process are described in Rosenbergová et al. [39]. Briefly, the microorganism was cultivated on YPD (*P. pastoris*) plates with 2% (*w*/*v*) agar and 50 mg/L ZeocinTM (InvivoGen, San Diego, CA, USA). For flask cultivations of *P. pastoris*, BMGY [Buffered Glycerol-complex Medium; 1% (*w*/*v*) yeast extract, 2% (*w*/*v*) peptone, 1.34% (*w*/*v*) YNB, 4 × 10^−5^% (*w*/*v*) biotin, 1% (*v*/*v*) glycerol, and 100 mM potassium phosphate (pH 6)] and BMMH [Buffered Minimal Methanol Medium; 1.34% (*w*/*v*) YNB, 4 × 10^−5^% (*w*/*v*) biotin, 0.5% (*v*/*v*) methanol, and 100 mM potassium phosphate (pH 6)] media were used.

Gene coding for *Sl*Xyn30A (GenBank: ANB12318.1) was codon-optimized for *P. pastoris* and purchased from Generay Biotech Co., Ltd. (Shanghai, China). Plasmid pPICZαA with ligated *xyn30A* was linearized with SacI (Fast Digest, ThermoFisher Scientific, Waltham, MA, USA). Approximately 5 µg of linearized plasmid was electroporated to *P. pastoris* KM71H competent cells (prepared according to Lin-Cereghino et al. [40]). Transformed cells were plated on YPD with 100, 150, 200 and 250 mg/L of ZeocinTM and cultivated at 30 °C for 48 h.

Four transformants from YPD plates with 250 mg/L of ZeocinTM were selected and screened for a recombinant *Sl*Xyn30A production. Additionally, 500 mL shake-flasks with 100 mL of BMGY medium were inoculated with a single *P. pastoris* colony and cultivated at 30 °C and 200 rpm for 22 h. The induction of enzyme was carried out as reported previously [41]. The cells were harvested by centrifugation (7197× *g*, 10 °C, 5 min), resuspended in 6 mL of sterile distilled water and transferred to 100 mL of BMMH medium with 0.5% (*v*/*v*) methanol. The cells were then cultivated at 30 °C and 200 rpm for 120 h, and methanol (100 µL) was added 2 times per day. After termination of cultivation, biomass was centrifuged (7197× *g*, 10 °C, 5 min) and the supernatants were concentrated and desalted on Microcon centrifugal filter devices (10 kDa cut-off, Millipore) and used for enzyme characterization. Protein concentration was determined by Bradford method using BSA as a standard [42].

### 4.4. Determination of pH and Temperature Optimum and Temperature Stability

pH optimum was determined at 50 °C using 10 mg·mL^−1^ solution of GX in 40 mM Britton–Robinson buffer (pH 2.0–8.0), 15 min incubation, and 64.4 nM *Sl*Xyn30A. Temperature optimum was determined in the same manner in 50 mM sodium acetate buffer, pH 3.5, and temperatures ranging from 23 °C to 60 °C. Temperature stability was tested in 50 mM sodium acetate buffer, pH 3.5, at 40–60 °C for up to 5 h. During the incubation, aliquots were taken at different time points and the residual activity was immediately determined as described above (10 mg·mL^−1^ GX, pH 3.5, 50 °C, 15 min).

### 4.5. Hydrolysis of Polysaccharides and Oligosaccharides

For specific activity determination, solutions (10 mg·mL^−1^) of GX, Rho and AraX in 50 mM sodium acetate buffer, pH 3.5, were mixed with 64.4 nM (GX and Rho) or 644 nM (AraX) *Sl*Xyn30A and incubated at 50 °C. At time intervals (5–30 min) 100 µL aliquots were taken for a determination of released reducing sugars by Somogyi–Nelson procedure [43]. One unit of activity is defined as the amount of the enzyme liberating in 1 min 1 μmol of reducing sugars expressed as an equivalent of xylose. All reactions were performed at least in triplicate. Kinetic parameters for GX hydrolysis were determined at 50 °C in 50 mM sodium acetate, pH 3.5, using 2.5–30 mg·mL^−1^ substrate concentration and 64.4 nM *Sl*Xyn30A. Kinetic constants were calculated by a non-linear regression using Origin 6.0 program (OriginLab Corp., Northampton, MA, USA).

For TLC (thin-layer chromatography) analysis, the same polysaccharide solutions (GX, Rho, AraX) were incubated with 1.6 µM *Sl*Xyn30A at 50 °C. Aliquots of 5 µL were spotted on silica-gel-coated aluminum sheets (Merck, Darmstadt, Germany) after 2 min, 1 h, 5 h, and 24 h of hydrolysis of GX, and 1 h and 24 h of hydrolysis of Rho and AraX. The reaction was terminated after 24 h by heating the mixtures at 100 °C for 5 min. Subsequent treatment with β-xylosidase (1 U·mL^−1^) was performed overnight at 50 °C. TLC plates were developed twice in the solvent system ethyl acetate/acetic acid/2-propanol/formic acid/water 25:10:5:1:15 (*v*/*v*) and the sugars were visualized with orcinol reagent (0.5% orcinol in 5% sulphuric acid in ethanol). For TLC analysis of Xyl_4_, MeGlcA^3^Xyl_4_, Xyl_3_-NP and methyl glycosides hydrolysis, 2.5 mM substrates were incubated with 1.6 μM *Sl*Xyn30A at 50 °C. Aliquots of 2 µL were spotted on TLC plate after 5 min, 1 h, 5 h and 24 h of hydrolysis. The plate was developed in a solvent system of *n*-butanol/ethanol/water 10:8:5 (*v*/*v*), and the sugars were visualized with the orcinol reagent.

For HPLC analysis, 5 mM Xyl_3_–Xyl_6_ solutions in 50 mM sodium acetate buffer, pH 3.5, were incubated with 1.3 µM *Sl*Xyn30A at 50 °C. At time intervals, 10 μL aliquots were taken and heated at 95 °C for 5 min. The samples were mixed with acetonitrile (1:4) and analyzed on a chromatographic apparatus Dionex UltiMate 3000 UHPLC system (ThermoFisher Scientific, Germering, Germany) equipped with a solvent degasser, quaternary pump, autosampler and thermostatic column compartment coupled to a Corona Veo RS detector (Thermo Fisher Scientific, Germering, Germany). Data processing was carried out with Chromeleon 7.2 SR3 software (Thermo Fisher Scientific, Waltham, MA, USA). Nitrogen gas was supplied by Peak nitrogen generator and Peak air compressor (Peak Scientific Instruments Ltd., Inchinnan, Renfrewshire, Scotland). The CAD device settings was as follows: data collection was set to 50.0 Hz at a filter constant of 3.6 s, power function for response and signal correction was set to 1.00 and evaporator temperature was set to 60 °C. Chromatographic separation was conducted on ARION HILIC Plus column (100 Å, 3.0 μm, 150 mm × 4.6 mm) maintained at 30 °C. Mobile phase A consisted of 0.5% acetic acid adjusted to pH 6.97 with NH_4_OH (25%, NH_3_ water solution), and mobile phase B was 100% acetonitrile. The elution was isocratic at a flow rate of 0.5 mL·min^−1^ with a mixture of mobile phases A and B in a ratio of 30:70. Specific activities were determined on 1 mM Xyl_3_, Xyl_4_, MeGlcA^3^Xyl_4_ and MeGlcA^3^Xyl_3_ in 50 mM sodium acetate buffer, pH 3.5, at 50 °C using 1.3 µM *Sl*Xyn30A, and calculated on the basis of the amount of liberated Xyl_2_ (linear XOs, in the case of Xyl_4_ divided by two) or Xyl (MeGlcA^3^Xyl_4_ and MeGlcA^3^Xyl_3_).

### 4.6. MALDI-ToF MS

The hydrolysates were decationized by Dowex 50 (H+ form) and 1 µL was mixed with 1 µL of the matrix (1% solution of 2,5-dihydroxybenzoic acid in 30% acetonitrile) directly on MS target plate. After air-drying, the samples were analyzed by UltrafleXtreme MALDI ToF/ToF mass spectrometer (Bruker Daltonics, Bremen, Germany) operating in reflectron positive mode.

## 5. Conclusions

The first GH30 xylanase originating from a yeast has been cloned, expressed and characterized. The enzyme *Sl*Xyn30A from *S. lignohabitans* is a glucuronoxylanase with auxiliary xylobiohydrolase activity. In addition to hardwood glucuronoxylan, it efficiently depolymerizes linear β-1,3-β-1,4-xylan but not cereal arabinoxylan. Its amino acid sequence has the highest similarity to the fungal bifunctional GH30-7 enzymes *Tc*Xyn30B and *Tt*Xyn30A which also display glucuronoxylanase and xylobiohydrolase activities. Catalytic properties of *Sl*Xyn30A also resemble those of *Tc*Xyn30B and *Tt*Xyn30A including the recognition of MeGlcA side chain in the −2b subsite, no substitution of xylose occupying the subsite −1, and certain flexibility of decoration of xylopyranosyl unit bound in the −2a subsite. Further characterization of new xylanases from different yeast species will help us to reveal how the yeasts cope with xylan degradation in nature and to better evaluate their biotechnological potential. The crystal structure of *Sl*Xyn30A with appropriate ligands would improve our knowledge of how GH30-7 glucuronoxylanases/xylobiohydrolases switch between endo- and exo-activities which is not yet fully understood.

## Data Availability

The data presented in this study are available on request from the corresponding author.

## Supplementary Materials

The following supporting information can be downloaded online, Figure S1: Multiple sequence alignment of GH30 catalytic domain of *Sl*Xyn30A with selected GH30-7 xylanases, Figure S2: SDS-PAGE electrophoresis of recombinant *P. pastoris* fermentation broths after induction with methanol.

## Sample Availability

Samples of the compounds are available from the authors.

## Abbreviations

EX	endo-β-1,4-xylanase
XOs	xylooligosaccharides
GH	glycoside hydrolase
GX	4-*O*-methylglucuronoxylan
MeGlcA	4-*O*-methylglucuronic acid
AraX	arabinoxylan
Rho	rhodymenan (linear β-1,3-1,4-xylan)
Xyl*p*	xylopyranosyl
Xyl_n_	linear xylooligosaccharide composed of n d-xylopyranosyl residues linked by β-1,4-linkages
MeGlcA^z^Xyl_n_	Xyl_n_ wherein zth xylopyranosyl residue counted from reducing end is α-glycosylated at position 2 by MeGlcA
NP	4-nitrophenyl
YPD	yeast extract peptone dextrose
YNB	yeast nitrogen base
