# Supplementary material for: Yeast GH30 Xylanase from Sugiyamaella lignohabitans Is a Glucuronoxylanase with Auxiliary Xylobiohydrolase Activity
Source: Molecules. 2022 Jan 25;27(3):751. doi: 10.3390/molecules27030751 (PMC8840591; doi:10.3390/molecules27030751)
Supplement: Supplementary file 1 [file molecules-27-00751-s001.zip › molecules-1556607-supplementary.pdf]

Supplementary material for the following manuscript:

Yeast GH30 xylanase from *Sugiyamaella lignohabitans* is a glucuronoxylanase with auxiliary xylobiohydrolase activity

by Katarína Šuchová, Andrej Chyba, Zuzana Hegyi, Martin Rebroš and Vladimír Puchart.

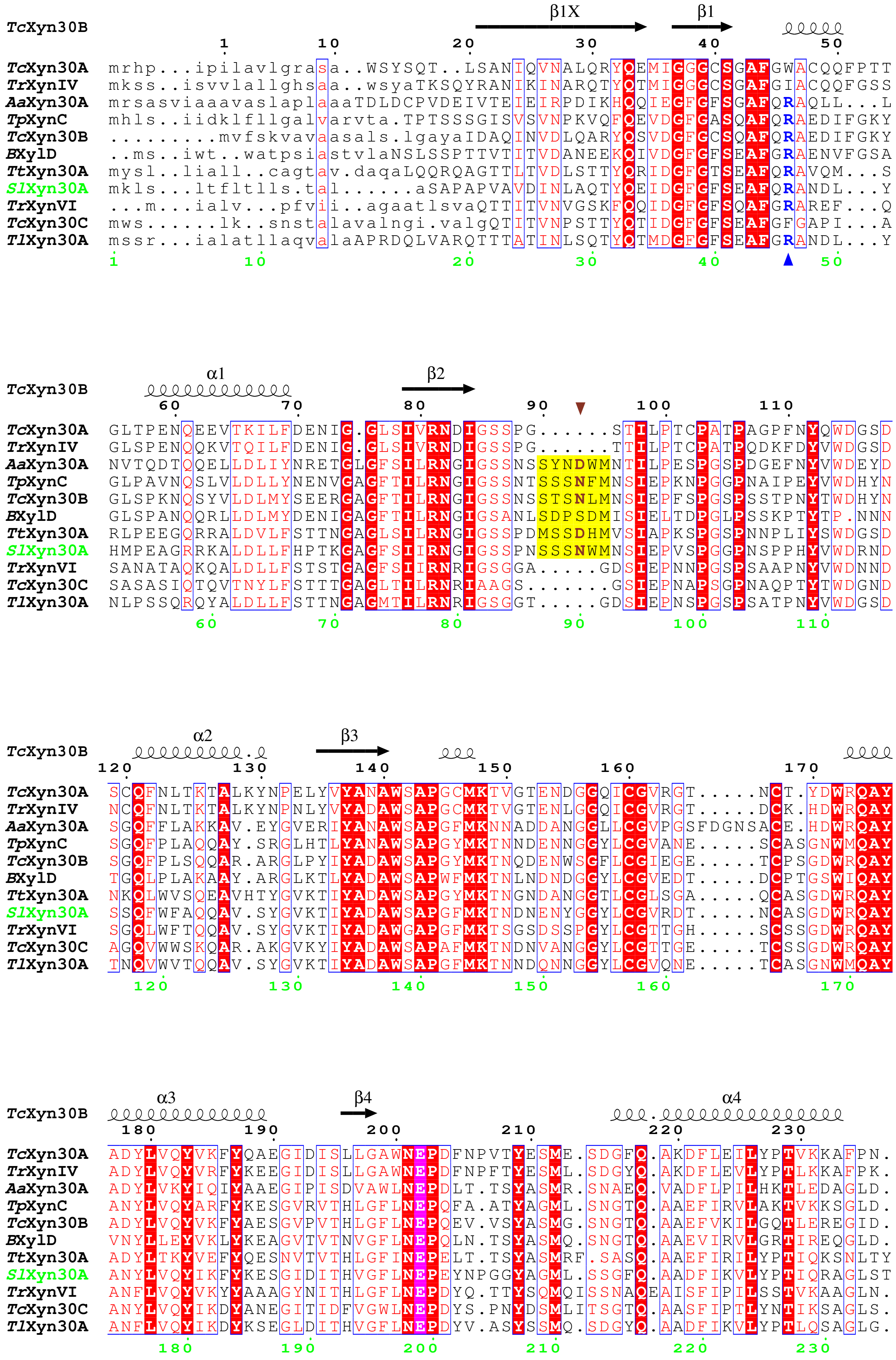
TcXyn30B β1X β1
1 10 20 30 40 50
TcXyn30A mrhp...ipilavlgrasa..WSYSQT..LSANIQVNALQRYQEMIGGGCSGAFGWACQQFPTT
TrXynIV mkss..isvvlallghsaa..wsyaTKSQYRANIKINARQTYQTMIGGGCSGAFGIACQQFGSS
AaXyn30A mrsasviaaavaslaplaaaTDLDCPVDEIVTEIEIRPDIKHQQIEGFGFSGAFQRAQLL...L
TpXynC mhls..iidklfllgalvarvta.TPTSSSGISVSVNPKVQFQEVDGFGASQAFQRAEDIFGKY
TcXyn30B ....mvfskvavaasals.lgayaIDAQINVDLQARYQSVDGFGCSQAFQRAEDIFGKY
BXylD ..ms..iwt..watpsiastvlaNSLSSPTTVTITVDANEEKQIVDGFGFSEAFGRAENVFGSA
TtXyn30A mysl..liall..cagtav.daqaLQQRQAGTTLTVDLSTTYQRIDGFGTSEAFQRAVQM...S
SlXyn30A mkls...ltfltlls.tal......aSAPAPVAVDINLAQTYQEIDGFGFSEAFQRANDL...Y
TrXynVI ...m...ialv...pfvii..agaatlsvaQTTITVNVGSKFQQIDGFGFSQAFGRAREF...Q
TcXyn30C mws......lk..snstalavalngi.valgQTITVNPSTTYQTIDGFGFSEAFGFGAPI...A
TlXyn30A mssr...ialatllaqvalaAPRDQLVARQTTTATINLSQTYQTMDGFGFSEAFGRANDL...Y
1 10 20 30 40 50

TcXyn30B α1 β2
60 70 80 90 100 110
TcXyn30A GLTPENQEEVTKILFDENIG.GLSIVRNDIGSSPG......STILPTCPATPAGPFNYQWDGSD
TrXynIV GLSPENQQKVTQILFDENIG.GLSIVRNDIGSSPG......TTILPTCPATPQDKFDYVWDGSD
AaXyn30A NVTQDTQQELLDLIYNRETGLGFSILRNGIGSSNSSYNDWMNTILPESPGSPDGEFNYVWDEYD
TpXynC GLPAVNQSLVLDLLYNENVGAGFTILRNGIGSSNTSSSNFMNSIEPKNPGGPNAIPEYVWDHYN
TcXyn30B GLSPKNQSYVLDLMYSEERGAGFTILRNGIGSSNSSTSNLMNSIEPFSPGSPSSTPNYTWDHYN
BXylD GLSPANQQRLLDLMYDENIGAGFTILRNGIGSANLSDPSDMISIELTDPGLPSSKPTYTP.NNN
TtXyn30A RLPEEGQRRALDVLFSTTNGAGLSILRNGIGSSPDMSSDHMVSIAPKSPGSPNNPLIYSWDGSD
SlXyn30A HMPEAGRRKALDLLFHPTKGAGFSIIRNGIGSSPNSSSNWMNSIEPVSPGGPNSPPHYVWDRND
TrXynVI SANATAQKQALDLLFSTSTGAGFSIIRNRIGSGGA.....GDSIEPNNPGSPSAAPNYVWDNND
TcXyn30C SASASIQTQVTNYLFSTTTGAGLTILRNRIAAGS.......GSIEPNAPSGPNAQPTYTWDGND
TlXyn30A NLPSSQRQYALDLLFSTTNGAGMTILRNRIGSGGT.....GDSIEPNSPGSPSATPNYVWDGSD
60 70 80 90 100 110

TcXyn30B α2 β3
120 130 140 150 160 170
TcXyn30A SCQFNLTKTALKYNPELYVYANAWSAPGCMKTVGTENDGGQICGVRGT.....NCT.YDWRQAY
TrXynIV NCQFNLTKTALKYNPNLYVYADAWSAPGCMKTVGTENLGGQICGVRGT.....DCK.HDWRQAY
AaXyn30A SGQFFLAKKAV.EYGVERIYANAWSAPGFMKNNADDANGGLLCGVPGSFDGNSACE.HDWRQAY
TpXynC SGQFPLAQQAY.SRGLHTLYANAWSAPGYMKTNNDENNGGYLCGVANE.....SCASGNWMQAY
TcXyn30B SGQFPLSQQAR.ARGLPYIYADAWSAPGYMKTNQDENWSGFLCGIEGE.....TCPSGDWRQAY
BXylD TGQLPLAKAAY.ARGLKTLYADAWSAPWFMKTNLNDNDGGYLCGVEDT.....DCPTGSWIQAY
TtXyn30A NKQLWVSQEAVHTYGVKTIYADAWSAPGYMKTNGNDANGGTLCGLSGA.....QCASGDWRQAY
SlXyn30A SSQFWFAQQAV.SYGVKTIYADAWSAPGFMKTNDNENYGGYLCGVRDT.....NCASGDWRQAY
TrXynVI SGQLWFTQQAV.SYGVKTIYADAWGAPGFMKTSGSDSSPGYLCGTTGH.....SCSSGDWRQAY
TcXyn30C AGQVWWSKQAR.AKGVKYIYADAWSAPAFMKTNDNVANGGYLCGTTGE.....TCSSGDWRQAY
TlXyn30A TNQVWVTQQAV.SYGVKTIYADAWSAPGFMKTNNDQNNGGYLCGVQNE.....TCASGNWMQAY
120 130 140 150 160 170

TcXyn30B α3 β4 α4
180 190 200 210 220 230
TcXyn30A ADYLVQYVKFYQAEGIDISLLGAWNEPDFNPVTYESME.SDGFQ.AKDFLEILYPTVKKAFPN.
TrXynIV ADYLVQYVRFYKEEGIDISLLGAWNEPDFNPFTYESML.SDGYQ.AKDFLEVLYPTLKKAFPK.
AaXyn30A ADYLVKYIQIYAAEGIPISDVAWLNEPDLT.TSYASMR.SNAEQ.VADFLPILHKTLEDAGLD.
TpXynC ANYLVQYARFYKESGVRVTHLGFLNEPQFA.ATYAGML.SNGTQ.AAEFIRVLAKTVKKSGLD.
TcXyn30B ADYLVQYVKFYAESGVPVTHLGFLNEPQEV.VSYASMG.SNGTQ.AAEFVKILGQTLEREGID.
BXylD VNYLLEYVKLYKEAGVTVTNVGFLNEPQLN.TSYASMQ.SNGTQ.AAEVIRVLGRTIREQGLD.
TtXyn30A ADYLTKYVEFYQESNVTVTHLGFINEPELT.TSYASMRF.SASQ.AAEFIRILYPTIQKSNLTY
SlXyn30A ANYLVQYIKFYKESGIDITHVGFLNEPEYNPGGYAGML.SSGFQ.AADFIKVLYPTIQRAGLST
TrXynVI ANFLVQYVKYYAAAGYNITHLGFLNEPDYQ.TTYSQMQISSNAQEAISFIPILSSTVKAAGLN.
TcXyn30C ANYLVQYIKDYANEGITIDFVGWLNEPDYS.PNYDSMLITSGTQ.AASFIPTLYNTIKSAGLS.
TlXyn30A ANFLVQYIKDYKSEGLDITHVGFLNEPDYV.ASYSSMQ.SDGYQ.AADFIKVLYPTLQSAGLG.
180 190 200 210 220 230

```
            β5          α5                      β6                     β7
TcXyn30B
             240       250       260       270       280       290
TcXyn30A   .LDVSCCDATGARQERNILYEVQQ.....AGGEHFFDVATWHNYQSSPERPFNVVGKPNIMTEW
TrXynIV    .VDVSCCDATGARQERNILYELQQ.....AGGERYFDIATWHNYQSNPERPFNAGGKPNIQTEW
AaXyn30A   .VGISCCEQTGWQATQQMVSQIQA.....LGVEHLLTTWAGHEYTSPINAPLD.TELSVWQSEY
TpXynC     .VKITCCDGIGWDDQEAMMAGLEA.GPD..PAINYLDVVTGHGYDSAPTYPLS.TNKKTWQSEW
TcXyn30B   .IELTCCDGVGWSEQEAMIPGLQVVGPDGKSAEDYLSVVTGHGYSSAPTFPLS.TKRRTWLTEW
BXylD      .LEINCCDAVGWEDAEQMLPGLQA.GPD..PAINYLSVYTGHGYASPPNFTLS.THLRTWLTEW
TtXyn30A   KPTIACCDAEGWNSQAGMLGALSS.......VNSMFGLVTAHAYTSQPGFSMN.TPHPVWMTEA
SlXyn30A   QVKIACCDSEGWDNQVNMTAEIQQ.....ENVEDLLGIITSHSYTSWPTYPIN.TKKNVWITES
TrXynVI    .TKLTCCDATGWTTQSTYTTNLVN.....AGSTQYLSVITSHSYSSDATSPLSQTSLPKWNTEG
TcXyn30C   .TGIACCDPFGWSDAVTWTAQLAS.....AGATQYLARITSHWYASKGTSPIN.TSLRVWETEY
TlXyn30A   NVGITCCDSMGWDSQKTRTQQLIS.....SGVENMLSVITSHSYSSDPTSSMG.TSLPVWQTEN
             240       250          260       270       280        290
```

```
                            α7              β8          β8A
TcXyn30B
           300       310       320       330       340       350
TcXyn30A   ADGSGPWNT.TWDVSGQLAEGLQWALYMHNAFTNSDTSGYNHWWCAGG.GADNV....LISITG
TrXynIV    ADGTGPWNS.TWDYSGQLAEGLQWALYMHNAFVNSDTSGYTHWWCAQNTNGDNA....LIRLDR
AaXyn30A   CDLNDRWTT.AWDSGVSRGDGYRWASVLHRALAVGNVNAYLWWLVVQDEATNNNNNEKLIMVED
TpXynC     ADLSGGFTPYDFYKNGGPGEGLTWANHIQVALRDAGVSGFLYWIGAENSTTNSG....LINLIN
TcXyn30B   TDLSGAFTPYTFFADGGAGEGMTWANHIQTAFVNANVSAFIYWIGAENSTTNSG....MINLIN
BXylD      ADLTGDYVPYVFFNNSGPGEGMTWANNIQVAFNAANVSAFLYWEGAENATASSS....LINLIN
TtXyn30A   ADLQGAWTS.AWYSYGGAGEGWTWANNVYNAIVNGNASAYLYWIGAQTGNTNSHM..VHIDANA
SlXyn30A   ADLNGLFAA.PWYNNSDAAEGLTWAQNLHTAIVDGHVSAYLYWEGVEVASSSSS...HFINVAN
TrXynVI    G.PSTPFVK.TWYSNGGTNEGFTWANKIAVAMVNAQLSAYLFWEGFEIQQSQSGS..HLIDALD
TcXyn30C   ADLDDAFTT.TWYSSGAANEGLTWANLIWQGVVEADLSAFLYWIGAQSNSNAAG....LVTL.N
TlXyn30A   ADLSDAWET.TWYSNGGAGEGMTWANKIYTAITEGGVSAYLYWEGIEDGTTNSC....LITI.N
              300       310       320       330       340        350
```

```
           β8B    α8            β2X        β3X        β4X        β5X
TcXyn30B
           360       370       380       390       400       410       420
TcXyn30A   NSY.EVSSRLWAFASYFRFARPGSVRIGATSSVENVYVSAYENKNGTVSIPVINAAHFPYEV.T
TrXynIV    DSY.EVSARLWAFAQYFRFARPGSVRIGATSDVENVYVTAYVNKNGTVAIPVINAAHFPYDL.T
AaXyn30A   GEYF.TSKRAWAFAHYSRYIRPDAYRVETRGG..DLSTTAYENKDGSLVVVILNPHHHSETVNL
TpXynC     NEVI.PSKRFWSFAQFSRFVRPGARRIEAASTHANLTVSSFENKDGRLATQLINQGDSTIEA.S
TcXyn30B   DEVI.PSKRFWSMASFSKFVRPNAQRVKATSSDASVTVSAFENTNGVVAIQVINNGTSAASL.T
BXylD      NEIV.LSKRYWVFAQFSKFVRPGARRVVASSSASDVFVTSFKNSNGVIATQVLNNGTSTTTI.D
TtXyn30A   G.TVEPSKRLWALGQWSRFVRPGARRVAVSGASGSLRTAAFRNEDGSVAVVVINSGGDAAVN.V
SlXyn30A   GQPV.TSSIYWALAQFSRFIRPGAVRVGTSSGSSSLSTSAYQNKDGSLSVQIVNNGAATPIQ.L
TrXynVI    RQTATPSGIFWAFAMWSRYIRPGASRVATSGSLSNVIIGAFQNTDRSIVVVFTNSGTSAQSA.R
TcXyn30C   GSTVQASGTLWAFAMFSRFIRPDAVRISTSGSPSNVNVGAFKNADGSIVVAINNNG..NSE.T
TlXyn30A   GTTVIPSGRLWAFGQWSRYVRPGAVRVAISGSTSNLKFTAFKNTDGTVSVQVLNFNS..AAT.T
               360       370       380       390       400       410
```

```
                      β6X       β7X β7'X     β8X        β9X
TcXyn30B
                  430       440       450       460       470
TcXyn30A   IDLQGLKAR...KRVSTFLTDNSHNVTLMDQ.SELHGSVLKATVPPRAVQVFWLE........
TrXynIV    IDLEGIKKR....KLSEYLTDNSHNVTLQSR.YKVSGSSLKVTVEPRAMKTFWLEPQSTFAVI
AaXyn30A   KLISCKHKNTGWGKVTAWLTDEEHDIEEVQV.EVDDEGYVKAELPARGLMTFKAVAEE.....
TpXynC     VRITGL.KS.G.VQVRPYITNNDYDLEPLKAVLSKNGGIFQANVPARSMVTYVSV........
TcXyn30B   IDLGKTHKE.V.KKVVPWVTSNDYDLEEMSEIDVKH.NSFLASVPARSLTSFVTECE......
BXylD      LEINGSWGW.GNREAMMYITDNEFDFKQEPV.YMGSDGLYHGTIPARSLVSFITQ........
TtXyn30A   RLASSSSADQQPASAKAWATDNSR.AIEEIQ.ASFADGVATVNVPSRSMTTVVLYPAADA...
SlXyn30A   QVSSRFGKNNLGLNFKAYVTDNSQNIAPLAI.KRNFDGTLTVNVPDHSLVTVTDLC.......
TrXynVI    VSFSGFT....PTSAAAYVTDNSH.TFSTTS.AGLSGGAITVSVPSKGVVTVKLT........
TcXyn30C   ISLSGIT....ASKVSAYYMDSAVSSPSTFS.ATLNGGTVGGSLPARSMVTFVIT........
TlXyn30A   VNINGFS....ASSVTAYVTDNTSNGIATLS.ASVSGGVASASIPGYSMVTLVLS........
             420       430       440        450       460       470
```

Figure S1. Multiple sequence alignment of *Sl*Xyn30A and all characterized eukaryotic GH30_7 xylanases, namely *Talaromyces cellulolyticus Tc*Xyn30B (GAM36763.1), *Tc*Xyn30C (GAM40414.1), *Tc*Xyn30A (GAM43270.1), *Thermothelomyces thermophila Tt*Xyn30A (AEO55025.1), *Talaromyces purpureogenus* (*Penicillium purpurogenum*) *Tp*XynC (AKH40280.1), *Bispora* sp. XylD (*B*XylD; ADG62369.1), *Trichoderma reesei* XynVI (*Tr*XynVI; EGR45006.1), *T. reesei* XynIV (*Tr*XynIV; AAP64786.1), *Acremonium alcalophilum Aa*Xyn30A [7], and *Talaromyces leycettanus Tl*Xyn30A [10]. The *Sl*Xyn30A amino acid numbering is shown in green at the bottom. Secondary structure elements and amino acid numbering of *Tc*Xyn30B is shown on top. Catalytic acid/base and catalytic nucleophile (in *Sl*Xyn30A Glu199 and Glu292, respectively) are violet highlighted. Conserved arginine (Arg46) which takes a part in MeGlcA recognition is typed in blue and is marked by a blue triangle. Extended region within a β2-α2 loop that is presumably responsible for xylobiohydrolase activity is yellow highlighted. Within the extended region an aspartic acid or an asparagine (Asn90) is often found and is typed in brown and marked by a brown triangle. The Asx residue (*Tc*Xyn30B Asn93 and *Tt*Xyn30A Asp78) has been demonstrated to be hydrogen bonded to Xyl*p* moiety accommodated in the -2a subsite.

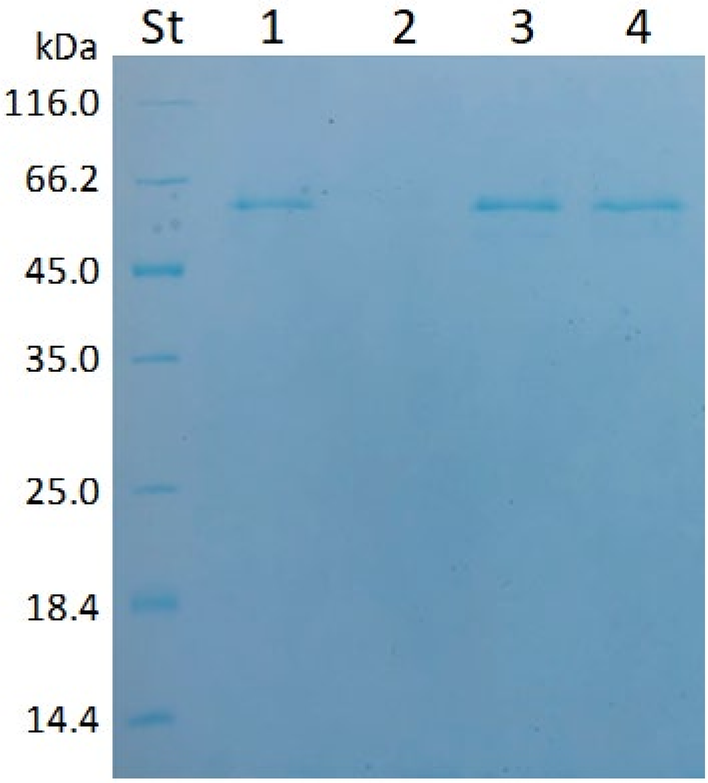

Figure S2: SDS-PAGE electrophoresis of recombinant *P.pastoris* fermentation broths after induction with methanol. St-MW standard, 1-4 fermentation broth of four transformants.
